# The contributions of nocturnal hypoxaemia and sleep disturbances to acute mountain sickness

**DOI:** 10.1113/EP093915

**Published:** 2026-08-18

**Authors:** D. M. Williamson, M. K. Gudelunas, J. W. Sall

**Affiliations:** ^1^ Department of Anesthesia and Perioperative Care University of California San Francisco San Francisco California USA

**Keywords:** acclimatization, acute mountain sickness, Lake Louise Scoring System, overnight oximetry, sleep architecture, sleep oximetry, sleep‐disordered breathing

## Abstract

Acute mountain sickness (AMS) is common after ascent to altitude, yet individual susceptibility remains difficult to predict and diagnosis relies on subjective report. Sleep disturbances and nocturnal hypoxaemia at altitude may provide insight, but their relationship to AMS is incompletely understood. In a sub‐study of a randomized, placebo‐controlled trial, 112 healthy, lowland‐dwelling volunteers rapidly ascended to 3810 m. AMS was assessed using the 2018 Lake Louise Scoring System (LLSS); sleep scores were recorded separately. Overnight monitoring used the WatchPAT300 device to measure oxygen saturation, oxygen desaturation index, respiratory disturbance index, apnoea–hypopnoea index and inferred sleep stage. Thirty‐eight participants (34%) developed AMS by the 2018 LLSS criteria. Compared with unaffected individuals, those with AMS had lower mean nocturnal $S_{pO_{2}}$, greater cumulative time with $S_{pO_{2}}$ < 70%, and higher oxygen desaturation index and respiratory disturbance index on nights 2 and 3. Respiratory disturbance indices improved only in participants without AMS, while AMS‐positive subjects demonstrated persistent reductions in estimated deep sleep, and higher self‐reported sleep disturbance scores. A sensitivity analysis applying 1993 LLSS criteria identified 64 subjects (58%) with AMS, and strengthened between‐group differences in nocturnal $S_{pO_{2}}$, but not respiratory indices. In exploratory multivariable analysis, cumulative time with $S_{pO_{2}}$ < 70% and first‐night sleep disturbance score at altitude remained independently associated with AMS. In summary, nocturnal hypoxaemia was associated with AMS status under both 2018 and 1993 criteria, while failure of respiratory index normalization and impaired sleep architecture are associated with AMS persistence, suggesting a longitudinal signal of failed acclimatization. These findings support further examination of sleep‐related metrics in relation to AMS assessment.

## Abstract (highlights)

- **What is the central question of this study?**
  The relationship among sleep disturbances, nocturnal hypoxaemia and acute mountain sickness (AMS) is poorly understood and the 2018 Lake Louise Scoring System was revised to remove the sleep disturbance component: do nocturnal sleep metrics constitute a meaningful signal of AMS?
- **What is the main finding and its importance?**
  Nocturnal hypoxaemia is associated with AMS status and is captured by both objective and subjective measures of sleep disturbance, supporting the relevance of the sleep component in the 1993 scoring system. Prolonged sleep disturbances at altitude may represent a distinct signal of failed acclimatization and are not well captured by the current scoring metric.

## 1 INTRODUCTION

Ascent to altitude for both recreation and work has become increasingly common in recent years. While many individuals are able to tolerate ascent to altitude without issue, studies have reported that nearly half of those who travel to 3000 m above sea level will experience some degree of acute mountain sickness (AMS) (Croughs et al., 2014; Hackett & Roach, 2001; Maggiorini et al., 1990); an incidence as high as 75% has been reported in those attempting Kilimanjaro (5948 m) (Karinen et al., 2008). Symptoms can range from mild and inconvenient to potentially life‐threatening, with common manifestations including headache, nausea, fatigue, and dizziness. More severe forms of altitude illness, such as high‐altitude pulmonary oedema (HAPE) and high‐altitude cerebral oedema (HACE), represent medical emergencies requiring immediate descent (Hackett & Roach, 2001).

Predicting who will get AMS remains a significant challenge, as susceptibility is the result of the complex interplay of individual, environmental and situational factors that are only just now beginning to be understood. Diagnosing and defining AMS can also be challenging, given that it relies on patient reports of several subjective factors, which may be tolerated to varying degrees by different individuals. The underlying cause of AMS is the physiological response to reduced oxygen availability at altitude. In contrast to sea level, the lower partial pressure of oxygen at altitude results in hypoxia, a state of insufficient oxygen delivery to the body's cells and tissues. The symptoms of AMS are thought to arise from the cerebrovascular and inflammatory responses to hypobaric hypoxia, including vasodilation, increased cerebral blood volume, and increased sympathetic activity (Hackett & Roach, 2001). However, while hypoxia is the causative agent of AMS, the degree of hypoxia does not reliably predict the incidence or severity of symptoms (Guo et al., 2014; O'connor et al., 2004). The Lake Louise Scoring System (LLSS), first proposed in 1993, utilizes self‐reported scores of symptom intensity for five core symptoms: headache, GI upset, fatigue/weakness, dizziness and sleep disturbance (Roach, 1993). Ascent to altitude disrupts normal sleep architecture and worsens subjective sleep quality. Polysomnography studies have shown a reduction in slow‐wave sleep, accompanied by a concomitant increase in light sleep and arousals during the acute period of acclimatization (Johnson et al., 2010; Weil, 2004). Prior studies have also reported an association between nocturnal desaturation and development of AMS (Burgess et al., 2004; Erba et al., 2004). These changes have been hypothesized to be driven by periodic breathing and nocturnal hypoxaemia, leading to sleep fragmentation. However, the sleep disturbance metric was removed from the LLSS in 2018 in response to studies demonstrating inconsistent correlation between sleep and AMS (Roach et al., 2018); in two large studies, the sleep quality score was only weakly correlated with other AMS symptoms, and thus it has been suggested that poor sleep should be considered a separate phenomenon (Hall et al., 2014; MacInnis et al., 2013). The use of nocturnal oximetry as a method to predict AMS has also been reported previously (Erba et al., 2004; Joyce et al., 2024); however, studies have generally been small and have primarily examined the incidence of AMS during graded ascent to altitude. Here, we report our observations of the effects of rapid ascent to 3810 m on overnight oximetry, estimated sleep architecture, subjective sleep quality and its relationship to the incidence of AMS.

## 2 METHODS

### 2.1 Ethical approval

Study protocols were approved by the University of California, San Francisco Institutional Review Board (IRB) and conformed to the *Declaration of Helsinki* (IRB no. 21‐33603, trial registration at ClinicalTrials.gov NCT03552263). All participants provided written informed consent prior to initiation of the study.

### 2.2 Study participants

This prospective study was a sub‐study within the context of a larger, randomized, placebo‐controlled phase 3 pharmaceutical trial sponsored by Tasly Pharmaceuticals Inc. (unpublished), investigating the efficacy of AR36 in preventing AMS during rapid ascent to 3810 m (White Mountain Research Center, Barcroft Station, CA, USA). One hundred and twelve healthy, non‐smoking volunteers between the ages of 18 and 55 years were enrolled. Participants were randomized to one of three groups: high dose (300 mg), low dose (225 mg), or placebo, beginning on study day 1. On study day 3, subjects were transported from sea level to 3810 m by car over approximately 8 h.

Key inclusion criteria included primary residence at ≤1000 ft (305 m) elevation, no travel to altitudes >10,000 ft (3048 m) within 4 months prior to screening, and normal baseline haematology, biochemistry and urinalysis. Key exclusion criteria included any medical history of cardiovascular, cerebrovascular, respiratory (including obstructive sleep apnoea, prescription/use of CPAP/BiPAP), renal, hepatic or other major medical comorbidities, blood oxygen saturation of <95% at sea level, recent surgery, blood donation, or treatment with any medications other than oral contraceptives within 14 days of screening and during the study. For full inclusion and exclusion criteria, see Supporting information, Table S1.

### 2.3 AMS assessment

Subjects were assessed for AMS using the 2018 LLSS each morning and evening while at altitude. AMS was defined as a LLSS ≥ 3 with at least one point for headache (Roach et al., 2018). Per the 2018 recommendations, the sleep disturbance item was not included in the AMS classification score; sleep scores were recorded separately. Morning LLSS scores were used to determine the presence or absence of AMS on a given day.

Enrolled participants wore a WatchPat300 (Yalamanchali et al., 2013) during sleep at sea level the night before ascent and each night at altitude. Polysomnography metrics, including oxygen desaturation index, respiratory disturbance index, and apnoea–hypopnoea index, were recorded by the device. Lake Louise scores were recorded each morning, along with morning oxygen saturations.

### 2.4 Oximetry measurements

Non‐invasive arterial saturation ($S_{pO_{2}}$) was measured as spot checks (approximately 1 min per measurement) using a Masimo Radical 7 pulse oximeter (Masimo Co., Irvine, CA, USA) applied to the left index finger. Measurements were taken before meals and before sleep while at altitude, and averaged into a single daytime value for analysis, as no significant time of day trend was observed.

### 2.5 Overnight sleep monitoring WatchPat300

WatchPat300 devices were provided by Itamar Medical (Caesarea, Israel). Briefly, the WatchPAT is validated home sleep apnoea testing device that utilizes peripheral artery tonometry (PAT), pulse oximetry and actigraphy to determine respiratory disturbances during sleep. Apnoeic and hypopnoeic events are detected indirectly by measuring changes in peripheral artery volume as described previously (Yalamanchali et al., 2013), which has been validated against standard polysomnography in multiple studies (Ayas et al., 2003; Bar et al., 2003; Hedner et al., 2011; Kenny et al., 2007; Penzel et al., 2002, 2004; Pillar et al., 2002; Pittman et al., 2004).

Participants wore the device for one night at sea level to capture baseline measures, and on nights 1, 2 and 3 after ascent to altitude. Data were recorded continuously (1 Hz). Data collected from the device were used to calculate oxygen desaturation index, respiratory disturbance index and apnoea–hypopnoea index, as described previously (Yalamanchali et al., 2013). Similarly, changes in PAT signal amplitude, heart rate, and actigraphy are used to differentiate between wakefulness, REM and non‐REM sleep, according to a device‐specific software algorithm.

#### 2.5.1 Oxygen desaturation index

The oxygen desaturation index (ODI) was calculated by determining the number of desaturation events, defined as a decrease in $S_{pO_{2}}$ ≥ 3% lasting at least 10 s, divided by the total sleep time.

#### 2.5.2 Peripheral artery apnoea–hypopnoea index (pAHI) and respiratory disturbance index

Changes in PAT signals (proprietary PAT signal) in conjunction with changes in vital signs were used to score respiratory events, including both apneic and hypopneic events, as well as total events of respiratory disturbance. The number of events divided by total hours of sleep was then used to calculate Peripheral artery apnoea–hypopnoea index (pAHI) or peripheral respiratory disturbance index (pRDI), respectively.

#### 2.5.3 Sleep phase

PAT signal amplitude and fluctuations were used in conjunction with heart rate, $S_{pO_{2}}$ and actigraphy to infer light sleep, deep sleep and REM sleep.

### 2.6 Statistical analysis

All statistical analyses were performed using Prism 10 software (GraphPad Software, Boston, MA, USA) or with Python (version 3.12.0; Python Software Foundation, Wilmington, DE, USA). Continuous variables were compared between AMS‐positive and AMS‐negative subjects at each night using ANOVA (ordinary or mixed‐effects model); significant effects were followed by Tukey *post hoc* comparisons, with adjusted *P*‐values reported. Subjects were classified as AMS‐positive or AMS‐negative independently for each night based on whether LLSS criteria were met, except where otherwise specified for the exploratory regression analysis. Associations between variables were assessed using Spearman's rank correlation. We considered *P*‐values of 0.05 or less significant. For exploratory analyses of factors associated with AMS, univariate binary logistic regression was performed with AMS status designated by whether they met 2018 LLSS criteria on any night at altitude; odds ratios and 95% confidence intervals were calculated by the Wald method for univariate analysis, and profile likelihood for multivariate analysis. Variables with *P *< 0.10 in the univariate screen were then used in multivariate logistic regression analysis. Multiple linear regression was also performed, with peak AMS score as the continuous outcome.

## 3 RESULTS

### 3.1 Subject demographics

A total of 112 subjects were enrolled in the study (66 males and 46 females, age 31.9 ± 9.1 years). Data collection was complete for 84 subjects at sea level (night 0), 100 on night 1 at altitude, 91 on night 2, and 83 on night 3; data attrition was primarily due to missed data collection in the primary study, or device issues (software or probe displacement), and did not differ between groups. Baseline sleep disturbance was minimal, with most participants reporting no sleep disturbance (72.5%), with 20.9% reporting a score of 1, and 6.6% reporting a score of 2. Subjects who developed AMS and those who did not were well matched at baseline, with no significant differences in age or sex between groups (age *P* = 0.098, sex *P* = 0.840). Sea level $S_{pO_{2}}$ and baseline nocturnal variables were also not significantly different between groups (Table 1).

**TABLE 1:** Participant characteristics and physiological variables by AMS status (2018 LLSS).

Variable	All subjects (*n* = 112)	AMS− (*n* = 73)	AMS+ (*n* = 38)	*P*
Age (years)	31.9 ± 9.1	32.7 ± 9.0	30.4 ± 9.4	0.098
Male, *n* (%)	66 (59%)	42 (58%)	23 (61%)	0.840
Female, *n* (%)	46 (41%)	31 (42%)	15 (39%)	0.840
Sea level $S_{pO_{2}}$ (%)	98.4 ± 1.2 (*n* = 109)	98.3 ± 1.2 (*n* = 71)	98.7 ± 1.2 (*n* = 38)	0.790
Mean nocturnal $S_{pO_{2}}$ (%)	95.6 ± 1.0 (*n* = 86)	95.7 ± 0.9 (*n* = 55)	95.5 ± 1.0 (*n* = 31)	0.637
Min nocturnal $S_{pO_{2}}$ (%)	90.9 ± 5.0 (*n* = 86)	91.1 ± 4.8 (*n* = 55)	90.6 ± 5.4 (*n* = 31)	0.884
Min < 70% (min)	0.0 ± 0.0 (*n* = 86)	0.0 ± 0.0 (*n* = 55)	0.0 ± 0.0 (*n* = 31)	1.000
pRDI (events/h)	14.6 ± 17.0 (*n* = 86)	14.3 ± 16.5 (*n* = 55)	15.1 ± 17.8 (*n* = 31)	0.993
pAHI (events/h)	5.2 ± 8.3 (*n* = 86)	5.4 ± 8.6 (*n* = 55)	4.9 ± 7.9 (*n* = 31)	0.366
ODI (events/h)	1.8 ± 4.0 (*n* = 86)	1.6 ± 3.3 (*n* = 55)	2.2 ± 4.8 (*n* = 31)	0.229
REM (%)	22.5 ± 9.0 (*n* = 79)	23.0 ± 10.2 (*n* = 51)	21.6 ± 6.8 (*n* = 28)	0.583
Light (%)	56.4 ± 11.8 (*n* = 79)	57.2 ± 12.6 (*n* = 51)	55.2 ± 10.5 (*n* = 28)	0.442
Deep (%)	21.9 ± 6.8 (*n* = 79)	21.1 ± 6.3 (*n* = 51)	23.2 ± 7.4 (*n* = 28)	0.179

*Note*: Data are means ± SD (*n* with available data) unless stated. Sleep architecture values are device‐estimated (WatchPAT300).

Abbreviations: ODI, oxygen desaturation index; pAHI, peripheral apnoea–hypopnoea index; pRDI, peripheral respiratory disturbance index.

### 3.2 AMS scores

**FIGURE A1:**
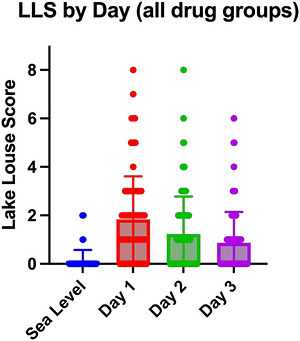
LLS by day. Lake Louise scores by day for all subjects (means ± SD, dots represent individual values).

**FIGURE A2:**
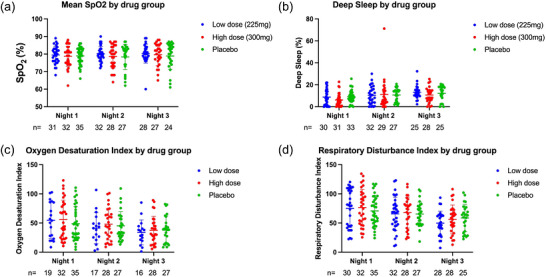
Effect of study drug on and sleep. (a) Mean night‐time $S_{pO_{2}}$ by night at altitude and study drug group; all results were non‐significant. (b–d) Percentage deep sleep (b), ODI (c) and RDI (d) by night and study drug group; all results were non‐significant.

A total of 38 subjects (34%) met 2018 LLSS criteria for AMS during the 3 days at altitude, with the highest incidence on night 1 (Appendix, Figure A1). No participants required medical intervention or were withdrawn from the study due to the severity of AMS symptoms. Incidence of AMS was not significantly different between the study drug and control groups (Supporting information, Table S2) (Appendix, Figure A2).

### 3.3 Peripheral oxygen saturation

#### 3.3.1 Daytime oximetry

**FIGURE 1:**
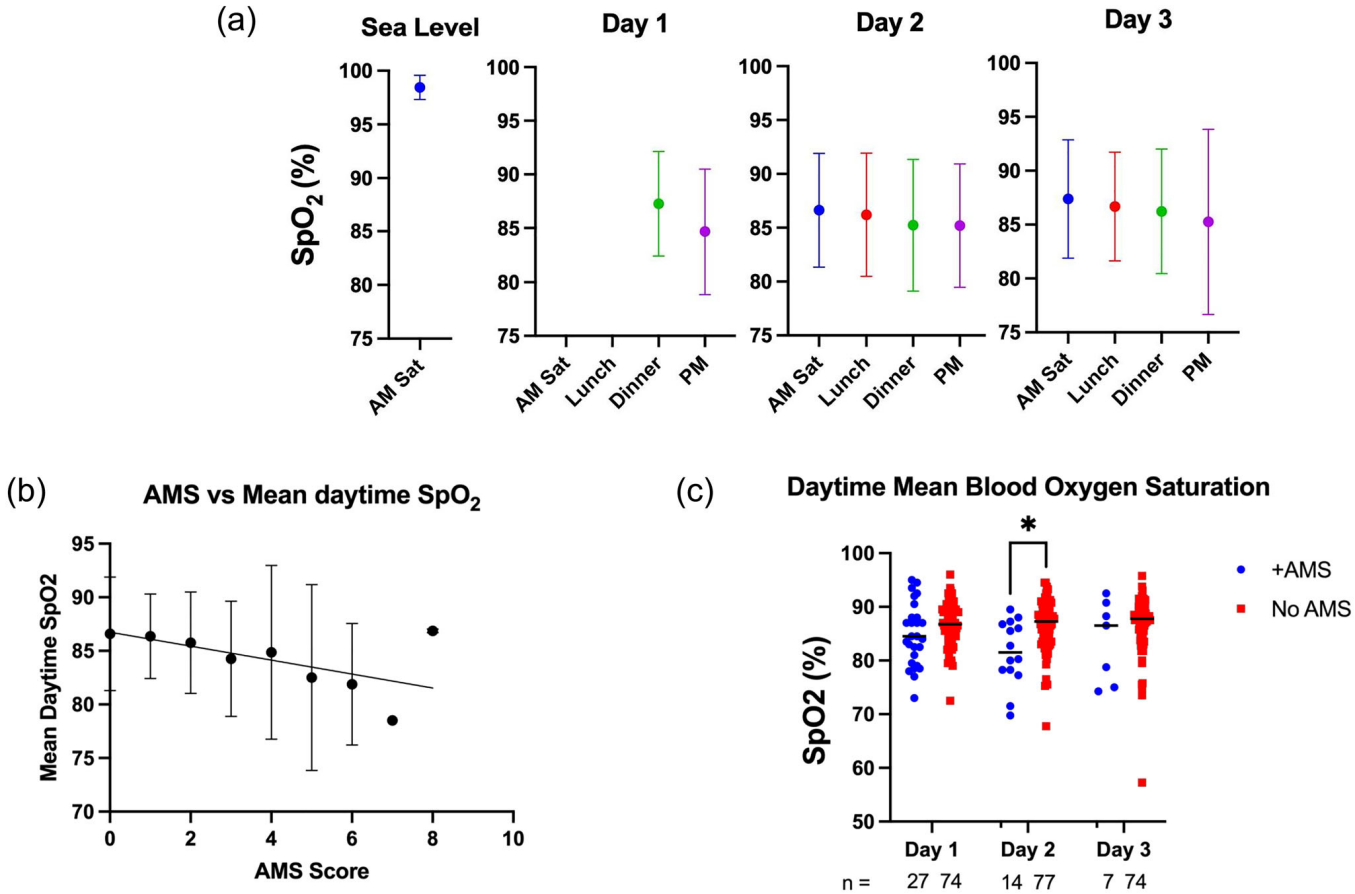
Daytime oxygen saturation. (a) Average $S_{pO_{2}}$ (mean ± SD) at each time point per day for all subjects (*n* = 112). (b) Association of mean daytime $S_{pO_{2}}$ and AMS score for all subjects on three days at altitude (Spearman's ρ = −0.21, *P* = 0.0005; *n* = 271) (c). Mean daytime saturation of subjects either meeting criteria LLSS for AMS or not by day. * *P* < 0.05.

Daytime saturations trended downward throughout the day, but this difference was not statistically significant (Figure 1a); therefore, measures were averaged into a single daytime value. Lower mean daytime $S_{pO_{2}}$ correlated with higher AMS scores (Figure 1b) (Spearman ρ = −0.21, *P* = 0.0005, *n* = 271); however, the differences between individuals that met LLSS criteria for AMS versus those who did not were only statistically significant on day 2 at altitude (Figure 1c, *P* = 0.0106), consistent with the well‐documented modest predictive value of resting daytime $S_{pO_{2}}$ for AMS (Guo et al., 2014; O'Connor et al., 2004).

#### 3.3.2 Overnight oximetry

**FIGURE 2:**
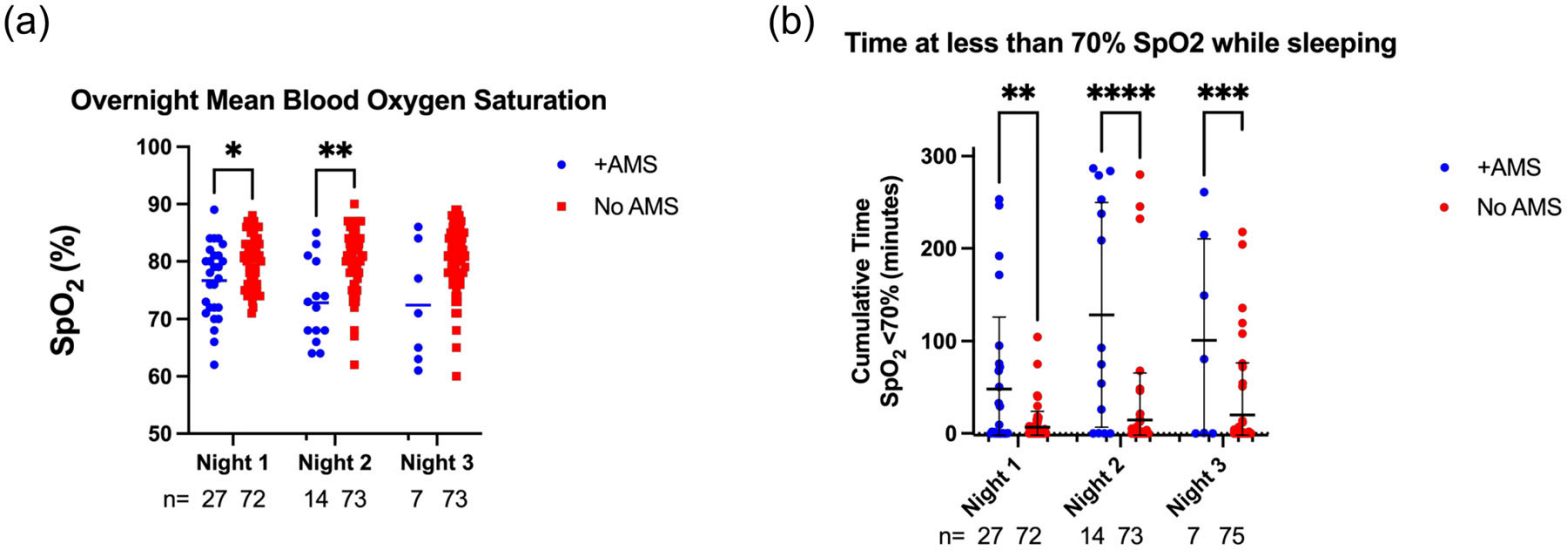
Overnight oxygen saturation. (a) Mean overnight $S_{pO_{2}}$ of subjects who either met LLSS criteria for AMS or not, by day. (b) Cumulative time (in minutes) with $S_{pO_{2}}$ < 70% for subjects who either met LLSS criteria for AMS or not, by night. *P < 0.05, **P < 0.005, ***P < 0.0005, ****P < 0.0001.

Participants who met criteria for AMS (LLSS ≥ 3) had lower night‐time mean $S_{pO_{2}}$ values on nights 1 and 2 at altitude than those who did not (night 1: 76.6 ± 6.4% vs. 80.0 ± 4.3%, *P* = 0.003; night 2: 72.9 ± 7.0% vs. 80.3 ± 5.1%, *P* < 0.001; Figure 2a). Cumulative time spent with $S_{pO_{2}}$ < 70% was greater in the AMS group on all three nights (48.1 ± 77.9 vs. 6.7 ± 17.1 min, *P* < 0.001; 128.3 ± 121.5 vs. 14.4 ± 51.1 min, *P* < 0.001; and 100.9 ± 109.4 vs. 19.9 ± 56.4, *P* = 0.001, respectively; Figure 2b).

#### 3.3.3 Respiratory disturbance indices

**FIGURE 3:**
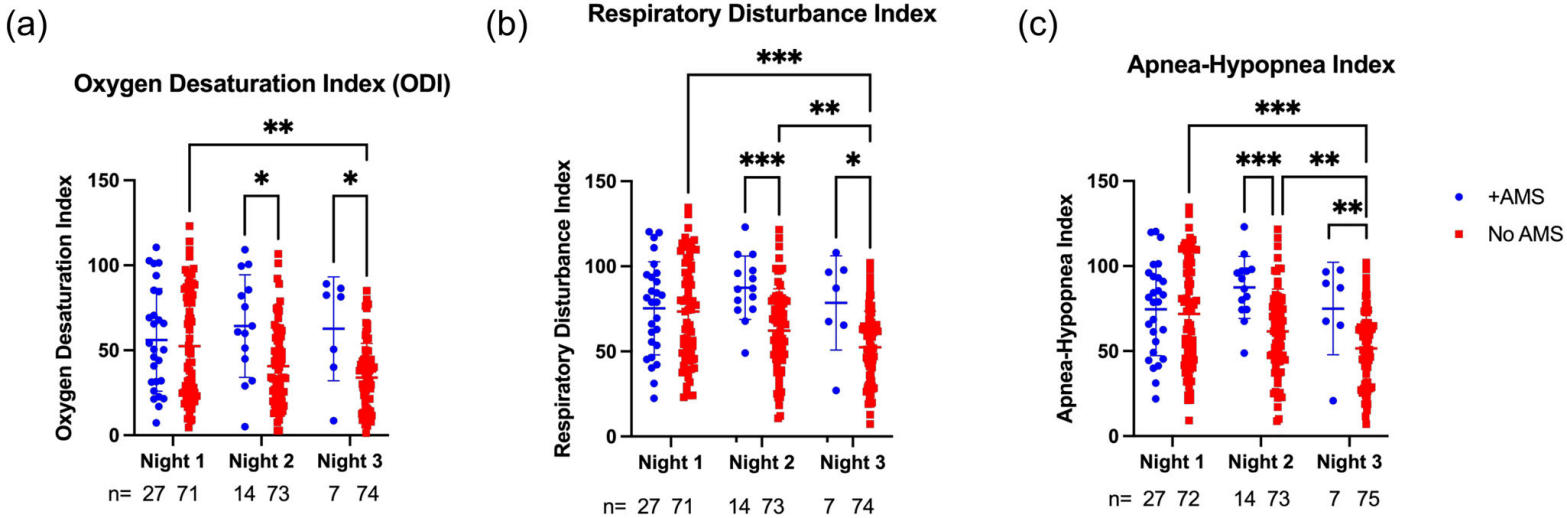
Overnight oximetry. Oxygen desaturation index (a), respiratory disturbance index (b), and apnoea–hypopnoea index (c) for subjects who met criteria for AMS or not, by night at altitude. *P < 0.05, **P < 0.005, ***P < 0.0005, ****P < 0.0001.

Oxygen desaturation index (ODI; Figure 3a) and respiratory disturbance index (RDI; Figure 3b) were higher on nights 2 and 3 at altitude in subjects with AMS relative to those without (ODI night 2, 64.3 ± 30.2 vs. 40.9 ± 23.8, *P* = 0.002; ODI night 3, 62.6 ± 30.5 vs. 34.0 ± 20.1, *P* < 0.001; RDI night 2, 87.5 ± 18.6 vs. 62.2 ± 24.6 *P* < 0.001; RDI night 3, 78.5 ± 27.7 vs. 52.5 ± 21.2, *P* = 0.003). Notably, both ODI and RDI improved by night 3 in subjects without AMS, whereas this improvement was not seen in subjects who met criteria for AMS. A similar trend was seen for the apnoea–hypopnoea index (AHI), which improved over time in non‐AMS subjects but remained unchanged in the AMS group (Figure 3c).

### 3.4 Estimated sleep architecture

**FIGURE 4:**
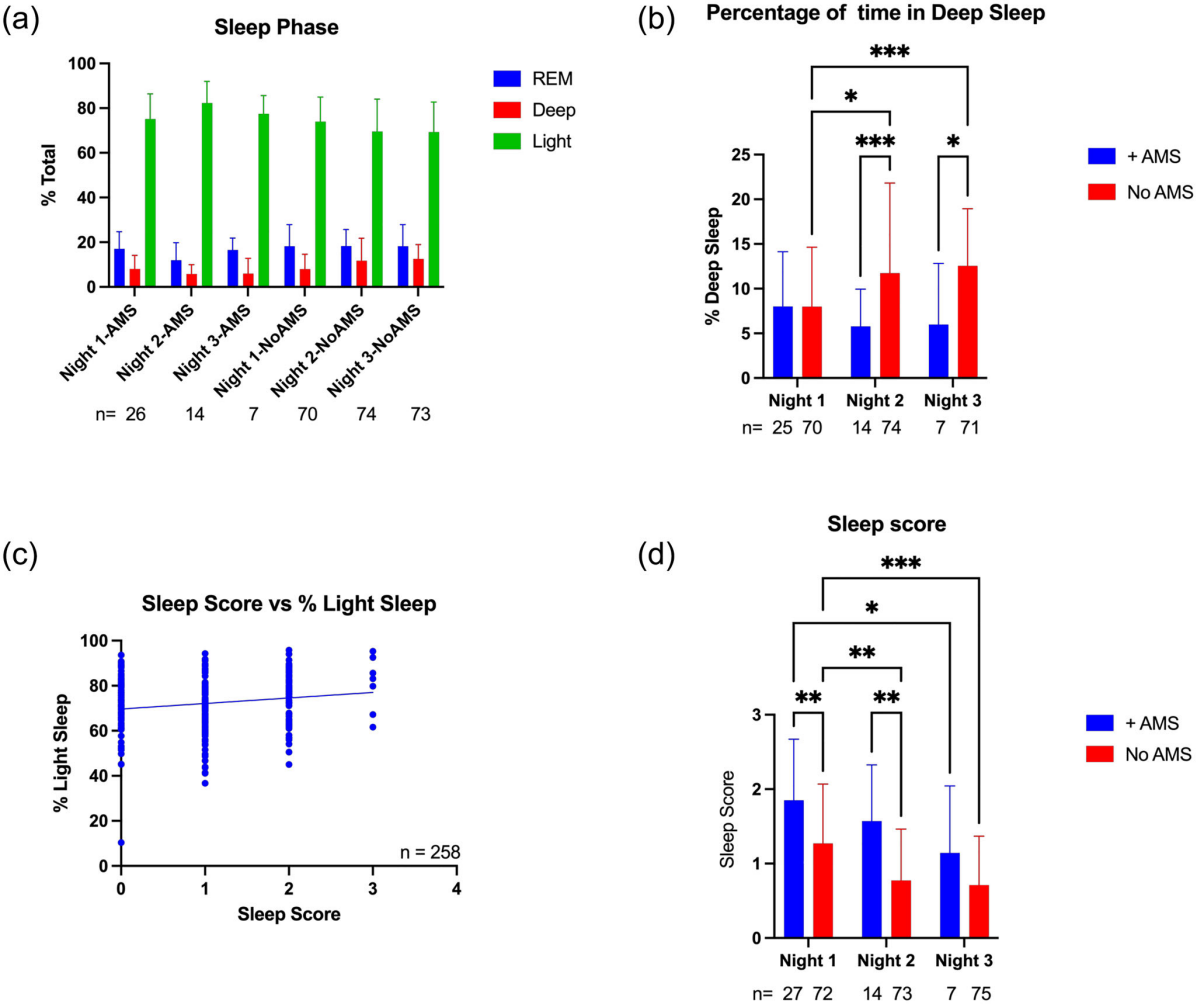
Sleep architecture. (a) Percentage of time spent in each phase of sleep by night across subjects meeting criteria for AMS versus those who did not. (b) Percentage of time spent in deep sleep during each night at altitude compared between subjects meeting criteria for AMS and those who did not. (c) Percentage of time spent in light sleep versus self‐reported sleep score across all subjects (*n* = 258). (d) Self‐reported sleep scores each night at altitude in subjects who met criteria for AMS versus those who did not. * *P* < 0.05, **P < 0.005, ***P < 0.0005.

While all participants spent the majority of sleep time in light sleep (Figure 4a), in subjects without AMS, the proportions of estimated deep and REM sleep increased with successive nights at altitude. In contrast, subjects who developed AMS spent less time in deep sleep on nights 2 and 3 (5.8% vs. 11.7%, *P* = 0.033 and 6.0% vs. 12.5%, *P* = 0.012; Figure 4b). We also noted a weak correlation (ρ = 0.15, *P* = 0.015, *n* = 258) between self‐reported sleep scores and percentage of time in light sleep across all subjects, where subjects who spent a greater portion of time in light sleep reported poorer sleep (i.e., higher sleep scores) (Figure 4c). Importantly, subjects with AMS reported statistically higher sleep disturbance scores on nights 1 and 2 (*P* = 0.0026 and 0.0018, respectively) (Figure 4d).

### 3.5 Sensitivity analysis: 2018 versus 1993 LLSS criteria

To evaluate the contribution of sleep disturbance to AMS, we performed a sensitivity analysis using the original 1993 LLSS criteria, which incorporates the subjective sleep disturbance score. Under the 1993 criteria, 64 subjects (58%) met criteria for AMS. Agreement between classification systems was 77% on night 1, 87% on night 2, and 93% on night 3, with the sleep score's contribution diminishing over time (Table 2). Mean night‐time $S_{pO_{2}}$ was lower and cumulative time with $S_{pO_{2}}$ < 70% higher in the AMS group under both scoring systems, but between‐group differences were strengthened using 1993 criteria.

**TABLE 2:** Sensitivity analysis: AMS group comparisons by 2018 and 1993 LLSS criteria.

Variable	2018: AMS−	2018: AMS+	*P* (2018)	1993: AMS−	1993: AMS+	*P* (1993)	Additional AMS+ under 1993
Night 1. Nocturnal oximetry							
**Mean nocturnal** $S_{pO_{2}}$ **(%)**	80.0 ± 4.3	76.6 ± 6.4	**0.003**	81.1 ± 4.1	77.1 ± 5.3	**< 0.0001**	*n* = 23
**Min nocturnal** $S_{pO_{2}}$ **(%)**	68.7 ± 6.7	64.1 ± 8.5	**0.007**	70.1 ± 6.3	64.8 ± 7.6	**0.0003**	*n* = 23
**Minutes** $S_{pO_{2}}$ ** < 70%**	6.7 ± 17.2	48.1 ± 77.9	**< 0.0001**	3.7 ± 11.6	32.0 ± 61.6	**0.002**	*n* = 23
**ODI (events/h)**	52.4 ± 32.0	56.1 ± 30.2	0.605	51.8 ± 34.1	55.0 ± 28.8	0.620	*n* = 23
**pRDI (events/h)**	73.5 ± 30.5	75.3 ± 27.4	0.783	72.7 ± 32.7	75.2 ± 26.4	0.680	*n* = 23
**pAHI (events/h)**	72.8 ± 30.9	74.5 ± 27.3	0.796	71.8 ± 33.3	74.6 ± 26.4	0.638	*n* = 23
**Deep sleep (%)**	8.0 ± 6.6	8.0 ± 6.1	0.989	8.5 ± 7.0	7.4 ± 5.9	0.411	*n* = 23
**REM sleep (%)**	18.0 ± 6.2	17.1 ± 7.7	0.525	18.4 ± 6.1	17.2 ± 7.1	0.377	*n* = 23
**Light sleep (%)**	74.0 ± 11.0	75.2 ± 11.2	0.634	73.0 ± 11.3	75.5 ± 10.7	0.274	*n* = 23
**LLSS score (2018 items only)**	1.0 ± 0.8	4.2 ± 1.5	**< 0.0001**	0.6 ± 0.8	3.0 ± 1.7	**< 0.0001**	*n* = 23
**Sleep score (0–3)**	1.3 ± 0.8	1.9 ± 0.8	**0.002**	1.1 ± 0.9	1.7 ± 0.7	**0.0001**	*n* = 23
**LLSS score (1993, inc. sleep)**	2.2 ± 1.3	6.0 ± 1.7	**< 0.0001**	1.8 ± 1.3	4.8 ± 1.9	**< 0.0001**	*n* = 23
**Mean daytime** $S_{pO_{2}}$ **(%)**	87.6 ± 4.5	82.5 ± 5.8	**0.0004**	88.2 ± 4.0	83.4 ± 5.9	**< 0.0001**	*n* = 12
**Mean nocturnal** $S_{pO_{2}}$ **(%)**	80.3 ± 5.1	72.9 ± 7.0	**< 0.0001**	81.2 ± 4.2	74.3 ± 7.1	**< 0.0001**	*n* = 12
**Min nocturnal** $S_{pO_{2}}$ **(%)**	68.9 ± 7.2	60.5 ± 9.2	**0.0003**	70.0 ± 6.7	61.8 ± 8.4	**< 0.0001**	*n* = 12
**Minutes** $S_{pO_{2}}$ ** < 70%**	14.4 ± 51.1	128.3 ± 121.5	**< 0.0001**	7.4 ± 36.6	92.2 ± 113.3	< **0.0001**	*n* = 12
**ODI (events/h)**	40.9 ± 23.8	64.3 ± 30.2	**0.002**	41.6 ± 24.3	51.9 ± 29.4	0.095	*n* = 12
**pRDI (events/h)**	62.2 ± 24.6	87.5 ± 18.6	**0.0005**	63.1 ± 25.1	73.8 ± 25.0	0.070	*n* = 12
**pAHI (events/h)**	61.6 ± 24.8	87.4 ± 18.3	**0.0004**	62.4 ± 25.4	73.7 ± 25.1	0.060	*n* = 12
**Deep sleep (%)**	11.7 ± 10.1	5.8 ± 4.2	**0.033**	11.8 ± 10.6	8.5 ± 6.1	0.142	*n* = 12
**REM sleep (%)**	18.3 ± 7.4	12.0 ± 7.8	**0.005**	17.5 ± 7.6	16.7 ± 8.4	0.662	*n* = 12
**Light sleep (%)**	69.5 ± 14.5	82.3 ± 9.7	**0.002**	70.1 ± 15.3	74.9 ± 12.4	0.161	*n* = 12
**LLS score (2018 items only)**	0.7 ± 0.8	4.1 ± 1.5	**< 0.0001**	0.5 ± 0.6	3.1 ± 1.5	**< 0.0001**	*n* = 12
**Sleep score (0–3)**	0.8 ± 0.7	1.6 ± 0.8	**0.0002**	0.7 ± 0.7	1.5 ± 0.6	**< 0.0001**	*n* = 12
**LLS score (1993, inc. sleep)**	1.5 ± 1.3	5.6 ± 1.8	**< 0.0001**	1.1 ± 1.1	4.5 ± 1.8	**< 0.0001**	*n* = 12
**Mean daytime** $S_{pO_{2}}$ **(%)**	87.6 ± 5.1	85.0 ± 8.4	0.236	88.0 ± 4.7	85.9 ± 7.1	0.183	*n* = 6
**Mean nocturnal** $S_{pO_{2}}$ **(%)**	80.3 ± 5.5	72.4 ± 10.1	**0.001**	80.9 ± 5.0	73.5 ± 9.0	**< 0.0001**	*n* = 6
**Min nocturnal** $S_{pO_{2}}$ **(%)**	70.6 ± 8.9	62.1 ± 11.5	**0.022**	71.2 ± 8.5	62.8 ± 10.8	**0.003**	*n* = 6
**Minutes** $S_{pO_{2}}$ ** < 70%**	19.9 ± 56.4	100.9 ± 109.4	**0.001**	15.4 ± 44.9	91.3 ± 113.5	**0.0001**	*n* = 6
**ODI (events/h)**	34.0 ± 20.1	62.6 ± 30.5	**0.0009**	35.5 ± 20.1	46.0 ± 30.8	0.124	*n* = 6
**pRDI (events/h)**	52.5 ± 21.2	78.5 ± 27.7	**0.003**	54.2 ± 20.9	62.9 ± 29.4	0.208	*n* = 6
**pAHI (events/h)**	51.6 ± 21.7	75.0 ± 27.2	**0.009**	53.2 ± 21.4	60.8 ± 28.3	0.272	*n* = 6
**Deep sleep (%)**	12.5 ± 6.4	6.0 ± 6.8	**0.012**	12.7 ± 6.7	9.2 ± 7.1	0.107	*n* = 6
**REM sleep (%)**	18.2 ± 9.7	16.6 ± 5.3	0.672	18.7 ± 10.0	16.9 ± 6.2	0.549	*n* = 6
**Light sleep (%)**	78.1 ± 76.6	77.5 ± 8.2	0.982	78.8 ± 81.8	73.9 ± 9.9	0.840	*n* = 6
**LLS score (2018 items only)**	0.6 ± 0.7	4.1 ± 1.2	**< 0.0001**	0.5 ± 0.7	3.1 ± 1.5	**< 0.0001**	*n* = 6
**Sleep score (0–3)**	0.7 ± 0.7	1.1 ± 0.9	0.120	0.6 ± 0.6	1.3 ± 0.9	**0.001**	*n* = 6
**LLS score (1993, inc. sleep)**	1.3 ± 1.2	5.3 ± 1.4	**< 0.0001**	1.1 ± 1.0	4.4 ± 1.5	**< 0.0001**	*n* = 6
**Agreement, Night 1**	—	—	—	77% (78/101)	—	—	23 reclassified
**Agreement, Night 2**	—	—	—	87% (77/89)	—	—	12 reclassified
**Agreement, Night 3**	—	—	—	93% (76/82)	—	—	6 reclassified
**Ever AMS+ (*n*, %)**	—	38 (34%)	—	—	64 (58%)	—	26 reclassified

Values are means ± SD. *P*‐values from two‐way repeated‐measures mixed‐effects model (REML) with AMS status and Night as fixed factors, Subject as random effect; within‐night AMS− vs. AMS+ comparisons by Tukey *post hoc* (adjusted *P*‐values). 2018 LLSS: score ≥3 + headache ≥1 (sleep excluded). 1993 LLSS: total score + sleep ≥3 + headache ≥1. Per‐night classification throughout.

Abbreviations: LLSS, Lake Louise Scoring System; ODI, oxygen desaturation index; pAHI, peripheral apnoea–hypopnoea index; pRDI, peripheral respiratory disturbance index.

In contrast, application of the 1993 LLSS criteria did not strengthen the between‐group differences in event‐based respiratory indices. Under the 2018 criteria, ODI, pRDI and pAHI were all significantly higher in subjects meeting criteria for AMS on nights 2 and 3; however, when the 1993 criteria were applied, the differences did not reach statistical significance on any night. Similarly, estimated deep sleep percentage was significantly lower in the group meeting criteria for AMS under the 2018 LLSS criteria on nights 2 and 3; when the 1993 LLSS criteria were used to distinguish between subjects with and without AMS, the differences in inferred sleep stage were not significant.

### 3.6 Exploratory analysis

#### 3.6.1 Univariate predictors of AMS status

To more robustly characterize factors associated with AMS, we performed univariate binary logistic regression for 39 candidate variables (demographic variables, daytime $S_{pO_{2}}$, nocturnal oximetry, respiratory disturbance indices, estimated sleep architecture, sleep disturbance scores) against binary AMS status (Supporting information, Table S3); subjects were classified using the ever‐AMS definition as a single composite outcome for prediction modelling, rather than the per‐night comparisons, which assess physiology specific to symptomatic nights. Neither age nor sex was associated with AMS status. Among altitude variables, significant associations (*P* < 0.05) were found for nocturnal $S_{pO_{2}}$ metrics on nights 1 and 3, cumulative time with $S_{pO_{2}}$ < 70% on nights 1 and 2, respiratory indices on night 2, and sleep score on night 1. Estimated sleep architecture was not significantly associated with the development of AMS on any night (Supporting information, Table S3).

#### 3.6.2 Multivariate logistic regression

We then performed multiple logistic regression analysis of the data with variables associated with AMS in the univariate analysis and deemed biologically relevant, with pAHI and ODI excluded as redundant with pRDI, and mean $S_{pO_{2}}$ excluded due to collinearity with threshold‐based desaturation metrics. Four variables were identified: cumulative time with $S_{pO_{2}}$ < 70% on nights 1 and 2, pRDI on night 2, and sleep disturbance score on night 1 (‘Model A’; *n* = 78, 24 AMS‐positive; Table 3). This model was significantly better than the null model (AICc 87.45 vs. 98.34, Δ = 10.89), well calibrated (Hosmer–Lemeshow *P* = 0.138), and had reasonable discriminant ability (area under the curve (AUC) = 0.758, 95% CI: 0.645–0.871). Adjusted odds ratios and 95% confidence intervals were estimated using the profile likelihood method. After adjustment, two variables remained significantly associated with AMS status: cumulative time with $S_{pO_{2}}$ < 70% on night 1 (odds ratio (OR) = 1.015, 95% CI: 1.002–1.034, *P* = 0.018), and sleep disturbance score on night 1 (OR = 2.088, 95% CI: 1.009–4.852, *P* = 0.048). Cumulative time with $S_{pO_{2}}$ < 70% on night 2 (*P* = 0.221) and pRDI on night 2 (*P* = 0.150) were not independently significant after adjustment. At a 0.5 probability threshold, sensitivity was 33.3% and specificity 94.4% (positive predictive value (PPV) 72.7%, negative predictive value (NPV) 76.1%); using Youden's optimal threshold (0.27), sensitivity increased to 81.5% and specificity decreased to 58.8%, suggesting greater utility for screening threshold. An analysis excluding pRDI (‘Model B’, *n* = 78, 24 AMS‐positive, Table 3) replicated these findings. A more parsimonious 2‐predictor model (‘Model C’, *n* = 99, 31 AMS‐positive), retaining only these two variables, confirmed both associations with similar effect sizes (OR 1.020, 95% CI: 1.008–1.039, *P* = 0.007, and OR = 2.197, 95% CI: 1.189–4.446, *P* = 0.021, respectively). Across all three models, sleep disturbance on night 1 demonstrated the most stable effect size, with an approximately two‐fold increase in AMS odds per unit increase in score.

**TABLE 3:** Multivariate logistic regression: Independent predictors of AMS status.

	Model A: 4‐predictor (univariate screen)			Model B: 3‐predictor (excl. pRDI N2)			Model C: 2‐predictor (parsimonious)		
Variable	OR	95% CI	*P*	OR	95% CI	*P*	OR	95% CI	*P*
**Min < 70%, Night 1 (min)**	**1.015**	1.002–1.034	**0.018**	**1.014**	1.000–1.029	**0.046**	**1.020**	1.008–1.039	**0.007**
**Min < 70%, Night 2 (min)**	1.005	0.998–1.014	0.221	1.005	0.998–1.013	0.168	—	—	—
**pRDI, Night 2 (events/h)**	1.019	0.996–1.045	0.150	—	—	—	—	—	—
**Sleep score, Night 1 (0–3)**	**2.088**	1.009–4.852	**0.048**	**2.049**	1.004–4.182	**0.049**	**2.197**	1.189–4.446	**0.021**
***n* (complete cases)**	**78**			**78**			**99**		
**AMS+ events**	**24**			**24**			**31**		
**Events/parameters**	**4.8**			**6.8**			**10.3**		
**Observations/parameters**	**15.6**			**19.5**			**33.0**		
**AICc – selected model**	**87.45**			**93.86**			**110.0**		
**AICc – null model**	**98.34**			**102.7**			**125.1**		
**AICc improvement (Δ)**	**10.89**			**8.82**			**15.10**		
**AUC**	**0.758**			**0.744**			**0.719**		
**AUC 95% CI**	**0.645–0.871**			**0.628–0.857**			**0.611–0.826**		
**Hosmer–Lemeshow *P***	**0.138**			—			**0.876**		
**All VIF < 5?**	**Yes (< 3.0)**			**Yes**			**Yes**		
**Sensitivity (cutoff 0.5)**	**33.3%**			—			**29.0%**		
**Specificity (cutoff 0.5)**	**94.4%**			—			**94.1%**		
**Sensitivity (Youden cutoff 0.27)**	**81.5%**			—			—		
**Specificity (Youden cutoff 0.27)**	**58.8%**			—			—		
**PPV/NPV (cutoff 0.5)**	**72.7%/76.1%**			—			**69.2%/74.4%**		
**CI method**	**Profile likelihood**			**Profile likelihood**			**Wald**		

*Note*: Model A: 4 predictors selected from univariate screen (*P *< 0.10); Model B: sensitivity analysis excluding pRDI N2. Model C: parsimonious primary model. Bold = *p* < 0.05, dash = variable not in model.

Abbreviations: AMS, acute mountain sickness; AUC, area under the curve; AICc, corrected Akaike information criterion; NPV, negative predictive value; PPV, positive predictive value; pRDI, peripheral respiratory disturbance index; VIF, variance inflation factor.

Finally, multiple linear regression was performed using the same four predictors with peak AMS score as the outcome. The resulting model was significant (*F*(4,73) = 8.80, *P* < 0.0001), with an *R* ^2^ = 0.325, and showed no evidence of multicollinearity (all variance inflation factor (VIF) <2; Supporting information, Table S4). Three variables were independently associated with AMS peak scores: cumulative time with $S_{pO_{2}}$ < 70% on night 1 (β = 0.0098, 95% CI: 0.002–0.018, *P* = 0.018), and night 2 (β = 0.0062, 95% CI: 0.001–0.011, *P* = 0.018), and subjective sleep disturbance score on night 1 (β = 0.562, 95% CI: 0.133–0.991, *P* = 0.011). pRDI on night 2 was not independently associated with peak AMS.

Notably, cumulative time with $S_{pO_{2}}$ < 70% on night 2 was independently significant in the linear model (severity of AMS), but not in the logistic model (presence of AMS), suggesting this variable is associated with how severe AMS becomes in those who develop it, rather than whether AMS occurs at all. Across logistic models A, B and C, cumulative time with $S_{pO_{2}}$ < 70% on night 1 and sleep disturbance on night 1 were the only variables that remained independently associated with AMS status in every specification, and were the strongest predictors of peak AMS score in the linear regression model. Respiratory disturbance indices and estimated sleep architecture were not associated with AMS severity in any model.

## 4 DISCUSSION

Here we report the relationship between nocturnal oximetry, sleep parameters and the development of AMS after rapid ascent to 3810 m. While the association between sleep desaturation and AMS has been established in prior work (Burgess et al., 2004; Erba et al., 2004), we believe the present study contributes several distinctive findings, particularly the longitudinal trajectory of respiratory disturbance indices across multiple nights, the independent predictive contributions of nocturnal hypoxaemia and subjective sleep disturbance after exploratory analysis, and the correspondence between objective and subjective sleep measures in the context of AMS.

The incidence of AMS in our cohort was highest on the first night at altitude, consistent with the well‐documented temporal pattern of AMS onset, which typically peaks within 6–24 h of ascent (Hackett & Roach, 2001). While daytime $S_{pO_{2}}$ levels were modestly lower in participants who developed AMS, differences in nocturnal oxygen saturation were more pronounced; subjects meeting LLSS criteria for AMS spent a greater cumulative time with $S_{pO_{2}}$ values below 70%, similar to previous studies (Burgess, Johnson, Edwards et al., 2004; Campbell & Sulaiman, 2023; Joyce et al., 2024). These periods of hypoxia and repetitive reoxygenation episodes impose stress through vascular and inflammatory mechanisms that contribute to characteristic AMS symptoms (Hackett & Roach, 2001; Maniaci et al., 2024).

A key finding of this study is the divergent trajectory of respiratory disturbance indices between subjects who developed AMS and those who did not over successive nights at altitude, observed with the 2018 LLSS classification. While all subjects experienced similar degrees of respiratory disturbance on the first night, ODI, RDI and AHI improved progressively in subjects without AMS, but remained persistently elevated in those who met 2018 AMS criteria. It is notable that these respiratory disturbances appeared to lag behind the peak in AMS incidence, consistent with prior studies demonstrating that periodic breathing persists even after oxygenation begins to improve (Nussbaumer‐Ochsner et al., 2012). This study extends that work by suggesting that the failure to acclimatize can be measured by degree of respiratory disturbance, and distinguishes AMS from non‐AMS subjects over time. Given the high altitude (>5000 m) threshold at which worsening of central sleep apnoea has been previously described, it is notable that this divergence was apparent at 3810 m.

Interestingly, the sensitivity analysis applying 1993 LLSS criteria substantially increased the AMS‐positive group size (from 34% to 58%), yet did not strengthen between‐group differences in respiratory indices (ODI, pRDI, pAHI). This dissociation reveals that the subjects reclassified as AMS‐positive under 1993 criteria – those whose sleep disturbance score alone pushed their total above threshold – had worse nocturnal $S_{pO_{2}}$ than the AMS‐negative group, but not a higher respiratory event burden. Because the 1993 classification incorporates sleep disturbance into the AMS definition, this enrichment for nocturnal hypoxaemic burden is not surprising, but is consistent with the altitude‐specific pattern of sustained rather than episodic desaturation: At 3810 m, a subject may accumulate considerable hypoxaemic stress with relatively few discrete events if their desaturations are prolonged and shallow rather than abrupt. Under these conditions, subjective sleep disturbance may capture the hypoxaemic burden that event‐counting indices miss, which explains why the 1993 classification selectively strengthens the nocturnal $S_{pO_{2}}$ signal. This interpretation is directly supported by the exploratory multivariate analyses: in binary logistic regression across all three model specifications, sleep disturbance on night 1 was independently associated with AMS status after adjustment for objective hypoxaemia, with the most parsimonious, two‐predictor model, which retained only cumulative time with $S_{pO_{2}}$ < 70% on night 1 and sleep disturbance score on night 1, confirming both associations with narrower confidence intervals. The independence of these two predictors suggests they capture distinct aspects of nocturnal physiology, and their combined performance provides strong quantitative evidence that subjective sleep disturbance and objective nocturnal hypoxaemia represent complementary, rather than redundant, signals of AMS risk. As above, the persistence of elevated event indices under 2018 criteria on nights 2 and 3 – in the group selected by four cardinal AMS symptoms – is therefore better understood as a marker of failed acclimatization rather than of initial hypoxaemic severity. Our data lack the temporal resolution to determine whether the respiratory disturbances were associated with periodic breathing or irregular, non‐periodic breathing, the latter of which has been reported to be more common in those who develop AMS (Weil, 2004).

Estimated sleep architecture, as inferred by the WatchPAT device, showed a related pattern of divergence between groups. While all participants spent the majority of time in light sleep, those without AMS demonstrated a progressive increase in deep and REM sleep with acclimatization, consistent with patterns described in polysomnography studies of altitude exposure (Johnson et al., 2010; Weil, 2004). In contrast, participants classified as AMS‐positive under the 2018 criteria did not have this shift in sleep phase, with a persistently lower proportion of estimated deep sleep on nights 2 and 3. These findings are consistent with prior data indicating that the fragmented sleep, increased arousals, and decreased slow‐wave (deep) sleep associated with ascent to altitude are particularly pronounced in those susceptible to AMS (Figueir et al., 2022; Nussbaumer‐Ochsner et al., 2012; Weil, 2004). The sensitivity analysis, unfortunately, does not cleanly extend this finding; subjects reclassified as AMS‐positive were predominantly from night 1 scores, and the small number with a persistent 1993‐only AMS‐positive classification on later nights precludes adequate power to examine architecture recovery in this subgroup. Furthermore, although we are unable to temporally correlate arousals to episodes of hypopnoea or apnoea, increases in objective measures of altered sleep architecture were associated with subjective reports of poor sleep, supporting an interaction between AMS and sleep quality. The exploratory multivariate analysis confirmed that among all nocturnal variables assessed, the duration of desaturation below 70% and subjective sleep quality on the first night, rather than sleep disturbance indices or sleep architecture, carry independent predictive information about AMS presence (logistic regression) and AMS severity (linear regression), supporting the utility of the sleep disturbance item as a surrogate marker of the underlying pathophysiological disturbance, even in the absence of formal polysomnography.

These findings have implications for the ongoing debate regarding the sleep component of the LLSS, and support a two‐pronged interpretation of the role of nocturnal physiology in AMS that requires careful distinction. First, nocturnal hypoxaemia is associated with AMS status; this association was present using the 2018 LLSS criteria, which excludes sleep disturbance. As reported above, AMS positive subjects had greater cumulative time with $S_{pO_{2}}$ < 70% than subjects without AMS on all three nights, and this was independently associated with AMS in the exploratory multivariate models. This indicates that the relationship between nocturnal hypoxaemia and AMS is independent of subjective sleep quality. However, incorporating subjective sleep disturbance into the LLSS classification further enriched the AMS‐positive group for individuals with greater nocturnal hypoxaemic burden, as evidenced by lower mean nocturnal $S_{pO_{2}}$ when the 1993 LLSS criteria were applied. This supports the role of the sleep component; in this context, subjective sleep disturbance may be understood as a correlate of nocturnal hypoxaemic burden, rather than an independent diagnostic component of AMS. This interpretation is consistent with the exploratory multivariate analyses suggesting that sleep disturbance score on night 1 carries independent predictive information about AMS presence and severity, even after controlling for objective oximetry, suggesting it captures physiology not fully reflected in $S_{pO_{2}}$ measurements alone. At the same time, subjective and objective measures are often discordant: It has been reported that sleep disturbance was absent in roughly 40% of cases of AMS, a cardinal symptom of AMS (Hall et al., 2014), consistent with the rationale for removing the sleep item from the 2018 revision of the scoring system. We suggest that the sleep question, although not perfectly correlated with other symptoms of AMS, may carry physiologically meaningful information about hypoxaemic burden that is not redundant with the other four symptoms. Secondly, our data suggest that failure to recover respiratory control and sleep architecture is a marker of AMS persistence, and is more robustly supported by the 2018 criteria; this may be due to the fact that the additionally captured subjects fall primarily in night 1, and acclimatize normally thereafter. Together, these data suggest that AMS is not monolithic from a nocturnal physiology standpoint: acute nocturnal hypoxaemia is associated with AMS status, and is captured by both objective $S_{pO_{2}}$ metrics and subjective sleep reporting, while failure of respiratory acclimatization is associated with symptom persistence.

Undoubtedly, there are numerous confounding factors that make the interpretation and incorporation of nocturnal physiology into AMS assessments challenging. In this study, poor sleep at altitude was common even in the absence of AMS, attributable to environmental factors, including sleeping in an unfamiliar setting, in dormitory‐style accommodation, and colder temperatures. It is worth noting that prior studies looking at the association of sleep quality, oximetry and AMS have varied in the altitude at which subjects sleep, with some studies including only daytime exposure or single exposures, short exposures in hypoxic chambers, or where subjects slept at altitudes lower than where symptoms of AMS were assessed. It is not surprising, therefore, that sleep scores have correlated poorly with other symptoms in these cases, and that removing the sleep question increases the internal consistency of the LLSS. Recent literature has suggested that the altitude at which one sleeps is often more important than the maximum altitude reached during the day (Horiuchi et al., 2018). We argue that our data, collected in participants sleeping at 3810 m for three consecutive nights, offers a more ecologically valid test of this relationship.

Taken together, we suggest that the removal of sleep from the LLSS may reflect methodological limitations rather than pathophysiological incongruence. The sleep question captures nocturnal hypoxaemia, the same biological signal that underlies AMS status, and thus its removal discards information that is particularly meaningful in studies where participants sleep at altitude for multiple consecutive nights. Future studies should consider both objective and subjective measures of nocturnal physiology, including continuous nocturnal $S_{pO_{2}}$ monitoring, as a practical, non‐invasive complement to subjective symptom‐based scoring that could improve risk stratification and acclimatization tracking in field settings. Evaluation of sleep quality may also be useful in providing guidance related to increasing activity or continued ascent, such as in winter sports athletes training at altitude camps, and in climbers planning progressive ascent.

### 4.1 Limitations

Several limitations should be considered when interpreting these findings. First, the WatchPAT is a home sleep apnoea testing device, not a full polysomnography system. Sleep stage classification is inferred from peripheral arterial tonometry, actigraphy and oximetry signals using a proprietary algorithm that has been validated in general clinical populations, but not specifically in high‐altitude environments, where peripheral vasoconstriction and alterations in autonomic tone may confound PAT‐based measurements. Similarly, the device does not include nasal airflow pressure transducers, thus AHI, ODI and RDI values may not conform precisely to American Academy of Sleep Medicine scoring criteria. Future studies incorporating full polysomnography at altitude are needed to validate these findings. Second, the use of an averaged daytime $S_{pO_{2}}$ may underestimate the variability or incidence of transient hypoxaemia, which could provide more nuanced data. With the increased availability, affordability and accuracy of wearable devices, future studies incorporating continuous daytime oximetry may provide additional insight into this aspect of altitude physiology. Similarly, no validated daytime sleep questionnaires were administered (e.g., Epworth Sleepiness Scale, Karolinska Scale, PSQI). The addition of these metrics would allow for a more comprehensive assessment of daytime functional impairment related to sleep disruption, and would complement the overnight WatchPAT data and LLSS sleep score.

While this study is one of the largest reported in subjects recruited from the general population, the number of subjects from whom full data were recorded was small, and the study was conducted at an altitude where incidence and severity of AMS is only expected to be moderate; future studies to target a larger number of subjects and excursions to higher altitudes are needed to further confirm these findings. The exploratory regression analyses were conducted in a sub‐study not originally powered for prediction modelling, and thus events‐per‐variable ratio in the logistic models were at or below conventional minimum thresholds. Similarly, the exploratory models include data from night 1, where incidence of AMS was also highest, and thus the predictors and outcome are potentially concurrent for a subset of subjects, rather than purely predictive of subsequent risk. Therefore, these analyses should thus be considered hypothesis‐generating, and require prospective confirmation in an adequately powered study, and with predictor variables assessed prior to outcome ascertainment to confirm whether these associations have true predictive value. Finally, these subjects were part of a larger investigational drug trial; thus, while our analysis did not show an effect of the drug on the sleep metrics we studied, we cannot exclude this possibility.

### 4.2 Conclusions

Nocturnal hypoxaemia is strongly associated with AMS following rapid ascent to altitude, and this association is strengthened when subjective sleep disturbance is incorporated into the AMS classification, as in the original 1993 LLSS. A sensitivity analysis demonstrated that subjects reclassified as AMS‐positive under 1993 criteria have lower nocturnal $S_{pO_{2}}$ than the 2018 AMS‐negative group, confirming that reported sleep disturbance captures pathophysiologically relevant hypoxaemic burden. Exploratory multivariate analyses further support that cumulative time with $S_{pO_{2}}$ < 70% and sleep disturbance score on the first night at altitude are independently associated with AMS presence and severity across multiple model specifications. The divergence in respiratory disturbance trajectory and estimated sleep architecture recovery between AMS and non‐AMS subjects, with progressive improvement in those without AMS but persistence in those with AMS, is most robustly supported under the 2018 classification and represents a potentially informative longitudinal signal that warrants further investigation. These two aspects of nocturnal physiology – acute hypoxaemia predicting symptoms, and failure of respiratory recovery predicting persistence – together argue for renewed consideration of sleep‐related nocturnal metrics in the assessment of AMS, particularly in the context of multi‐day altitude exposure. Overnight oximetry and device‐estimated sleep metrics offer a non‐invasive, practical approach to identifying individuals at risk and track acclimatization. Devices capable of tracking such metrics are rapidly becoming ubiquitous among those traveling to high altitudes for work and recreation and using such data may offer an objective rather than purely subjective assessment of the acclimatization process. Prospective studies adequately powered to test prediction models incorporating continuous nocturnal $S_{pO_{2}}$ and sleep disturbance assessment are needed to confirm these exploratory findings and define optimal thresholds for clinical use.

## AUTHOR CONTRIBUTIONS

M.K.G. and J.W.S. conceived and designed the study. All authors were involved in subject enrollment and data collection. D.M.W. performed data analysis and manuscript preparation. All authors revised the manuscript for content. All authors have read and approved the final version of this manuscript and agree to be accountable for all aspects of the work in ensuring that questions related to the accuracy or integrity of any part of the work are appropriately investigated and resolved. All persons designated as authors qualify for authorship, and all those who qualify for authorship are listed.

## CONFLICT OF INTEREST

The authors declare there are no potential conflicts of interest with respect to the research, authorship, and/or publication of this article.

## GENERATIVE AI STATEMENT

During the preparation of this manuscript, the authors used ChatGPT‐5.5 (UCSF Enterprise Version, OpenAI) for language editing to improve the clarity and readability of selected sections, and Claude Haiku 4.5 (Anthropic), accessed via ClaudeCode v2.1.101 within a Python IDE (Positron), to assist in writing Python code for exploratory univariate and multivariate analysis. All code and output were reviewed and verified.

## Supporting information



Appendix Table S1: Inclusion and Exclusion Criteria
Appendix Table S2: Incidence of AMS by day across all drug groups
Appendix Table S3. Univariate Logistic Regression: Predictors of AMS Status (2018 LLSS, Ever AMS+)
Appendix Table S4. Multiple Linear Regression: Independent Predictors of Peak AMS Scre

## Data Availability

Data that support these findings are available upon reasonable request from the corresponding author. Data are not publicly available for privacy reasons.
