# Supplementary material for: The contributions of nocturnal hypoxaemia and sleep disturbances to acute mountain sickness
Source: Exp Physiol. 2026 Aug 18:10.1113/EP093915. Online ahead of print. doi: 10.1113/EP093915 (PMC13487406; doi:10.1113/EP093915)
Supplement: Supplementary file 1 [file EPH-9999-0-s001.docx]

Appendix Table S1: Inclusion and Exclusion Criteria

Inclusion Criteria

1. Healthy volunteers: ages 18 - 55 years old;

2. Primary residence elevation of 1,000 ft or lower;

3. Not ascending to altitude >10,000 ft within 4 months prior to screening;

4. Females of childbearing potential must have a negative pregnancy test and established on a method of contraception that in the investigator’s opinion is acceptable. Females must agree to remain on their established method of contraception from the time of the screening visit and throughout the study period;

5. Willing to participate voluntarily and sign a written informed consent.

Exclusion Criteria

1. Subjects with medical history of cardiovascular, cerebrovascular diseases or asthma; uncontrolled hypertension with SBP>140 and or DBP>90 mmHg;

2. Subjects with current and clinically significant respiratory system disease, digestive disease, liver disease, central nervous system disease, psychiatric disease, metabolic disease, renal disease, acute infection or anemia;

3. Total LLSS self-assessment score is ≥2 at any check point during screening period;

4. Headache scores is greater than 3 assessed by VAS at any check points during screening period;

5. Blood oxygen saturation (SpO2) is less than 95% at screening visit;

6. Subjects with abnormal renal or liver function with clinical significance (ALT or AST > 2×ULN, Creatinine > ULN) at screening visit;

7. Subjects with CRP > ULN at screening visit;

8. Subjects with primary (migraine, tension-type headache, and cluster headache etc.) or secondary headaches (headache related to infection, vascular disease etc.) within one month at screening;

9. Surgery or blood donation within 3 months prior to screening;

10. On treatment of any medications (including any dietary supplements) except for birth control within 14 days prior to screening and throughout the study period;

11. Smokers who had a habit of smoking during the last 4 months prior to the starting of screening;

12. Contradictive to treatment of Danshen (Radix Saliva Miltiorrhize Bge., RSM) products;

13. Women in pregnancy or lactation period;

14. Substance abuse. Patients with a recent (within the last 2 years) history of substance abuse (alcohol, marijuana, or known drug dependence). Or patients who have a positive urine substance test at screening;

15.Participation in any other interventional clinical trial or on an investigational drug within 30 days prior to screening;

16. A family member or relative of the study site staff;

17. Any condition that, in the opinion of the investigator, is likely to prevent compliance with the study protocol, interfere with the assessment, or pose a safety concern if the subject participates in the study at screening.

Appendix Table S2: Incidence of AMS by day across all drug groups

|  | | **+ AMS (% of total)** | **- AMS** | **Total** |
| --- | --- | --- | --- | --- |
| Night 1 | Low | 7 (22.6) | 24 | 31 |
|  | High | 10 (29.4) | 24 | 24 |
|  | Placebo | 10 (28.6) | 25 | 35 |
| Night 2 | Low | 4 (11.8) | 30 | 34 |
|  | High | 6 (20) | 24 | 30 |
|  | Placebo | 4 (14.8) | 23 | 27 |
| Night 3 | Low | 0 (0.0) | 30 | 30 |
|  | High | 3 (10.7) | 25 | 28 |
|  | Placebo | 4 (16) | 21 | 25 |

Appendix Table S2: Number of subjects in each experimental drug group meeting criteria for AMS by night at altitude. Fisher’s exact test was used to compare the incidence of AMS in drug groups each day; all results were not significant. [ Day 1 p = 0.8483, Day 2 p =0.7142, Day 3 p=0.5353]

| **Appendix Table S3. Univariate Logistic Regression: Predictors of AMS Status (2018 LLSS, Ever AMS+)** | | | | |  |
| --- | --- | --- | --- | --- | --- |
| **Variable** | **n** | **OR** | **95% CI** | **p-value** |  |
| **DEMOGRAPHICS** | | | | |  |
| Age (years) | 111 | 0.971 | 0.928 – 1.017 | 0.215 |  |
| Sex (female vs male) | 111 | 0.884 | 0.397 – 1.965 | 0.761 |  |
| **DAYTIME SpO₂** | | | | |  |
| Daytime SpO₂, Day 1 (%) | 111 | 0.925 | 0.852 – 1.006 | 0.068 |  |
| Daytime SpO₂, Day 2 (%) | 111 | 0.959 | 0.890 – 1.032 | 0.264 |  |
| Daytime SpO₂, Day 3 (%) | 81 | 0.931 | 0.855 – 1.014 | 0.102 |  |
| **NOCTURNAL OXIMETRY — Night 1** | | | | |  |
| Mean nocturnal SpO₂ (%) | 99 | **0.909** | 0.835 – 0.990 | **0.028** |  |
| Min nocturnal SpO₂ (%) | 99 | **0.935** | 0.882 – 0.991 | **0.025** |  |
| Min <70% (min) | 99 | **1.019** | 1.004 – 1.035 | **0.015** |  |
| **NOCTURNAL OXIMETRY — Night 2** | | | | |  |
| Mean nocturnal SpO₂ (%) | 87 | 0.929 | 0.863 – 1.001 | 0.053 |  |
| Min nocturnal SpO₂ (%) | 87 | 0.950 | 0.898 – 1.005 | 0.072 |  |
| Min <70% (min) | 87 | **1.010** | 1.003 – 1.017 | **0.007** |  |
| **NOCTURNAL OXIMETRY — Night 3** | | | | |  |
| Mean nocturnal SpO₂ (%) | 80 | **0.922** | 0.854 – 0.995 | **0.036** |  |
| Min nocturnal SpO₂ (%) | 81 | 0.957 | 0.910 – 1.007 | 0.090 |  |
| Min <70% (min) | 82 | 1.006 | 0.999 – 1.013 | 0.115 |  |
| **RESPIRATORY INDICES — Night 1** | | | | |  |
| pRDI (events/hr) | 98 | 1.006 | 0.992 – 1.021 | 0.387 |  |
| pAHI (events/hr) | 97 | 1.006 | 0.992 – 1.021 | 0.392 |  |
| ODI (events/hr) | 98 | 1.008 | 0.995 – 1.022 | 0.225 |  |
| **RESPIRATORY INDICES — Night 2** | | | | |  |
| pRDI (events/hr) | 87 | **1.026** | 1.006 – 1.046 | **0.011** |  |
| pAHI (events/hr) | 87 | **1.026** | 1.006 – 1.046 | **0.010** |  |
| ODI (events/hr) | 86 | **1.023** | 1.005 – 1.042 | **0.012** |  |
| **RESPIRATORY INDICES — Night 3** | | | | |  |
| pRDI (events/hr) | 81 | 1.016 | 0.995 – 1.038 | 0.139 |  |
| pAHI (events/hr) | 81 | 1.015 | 0.994 – 1.037 | 0.165 |  |
| ODI (events/hr) | 81 | 1.016 | 0.995 – 1.037 | 0.142 |  |
| **SLEEP ARCHITECTURE — Night 1** | | | | |  |
| REM sleep (%) | 96 | 1.005 | 0.943 – 1.071 | 0.882 |  |
| Light sleep (%) | 96 | 1.003 | 0.965 – 1.042 | 0.884 |  |
| Deep sleep (%) | 95 | 0.991 | 0.928 – 1.060 | 0.801 |  |
| **SLEEP ARCHITECTURE — Night 2** | | | | |  |
| REM sleep (%) | 88 | 0.991 | 0.937 – 1.049 | 0.763 |  |
| Light sleep (%) | 88 | 1.019 | 0.985 – 1.053 | 0.274 |  |
| Deep sleep (%) | 88 | 0.968 | 0.914 – 1.024 | 0.259 |  |
| **SLEEP ARCHITECTURE — Night 3** | | | | |  |
| REM sleep (%) | 80 | 0.961 | 0.896 – 1.031 | 0.269 |  |
| Light sleep (%) | 80 | 1.037 | 0.991 – 1.085 | 0.114 |  |
| Deep sleep (%) | 78 | 0.940 | 0.870 – 1.015 | 0.113 |  |
| **SLEEP DISTURBANCE SCORE (0–3)** | | | | |  |
| Night 1 | 101 | **2.104** | 1.189 – 3.725 | **0.011** |  |
| Night 2 | 89 | 1.575 | 0.872 – 2.843 | 0.132 |  |
| Night 3 | 78 | 1.905 | 0.930 – 3.902 | 0.078 |  |
| **BASELINE (SEA LEVEL)** | | | | |  |
| Sea level mean nocturnal SpO₂ (%) | 86 | 0.881 | 0.545 – 1.426 | 0.607 |  |
| Sea level pRDI (events/hr) | 86 | 1.003 | 0.977 – 1.029 | 0.834 |  |
| Sea level deep sleep (%) | 79 | 1.048 | 0.976 – 1.125 | 0.195 |  |
| *OR = odds ratio. 95% CI by Wald method. Green=p<0.05. AMS outcome: Ever AMS+ (any night) by 2018 LLSS.* | | | | |  |
|  |  |  |  |  |  |

| **Appendix Table S4. Multiple Linear Regression: Independent Predictors of Peak AMS Score** | | | | | | | |  |
| --- | --- | --- | --- | --- | --- | --- | --- | --- |
| **Variable** | **β** | **95% CI** | **p** | **VIF** | **Model** | **n** | **R²** |  |
| **Primary Model — A priori selection (Prism; n=78, R²=0.322, F(4,73)=8.68, p<0.0001; all VIF<1.3)** | | | | | | | |  |
| Min <70%, Night 1 (min) | **0.0101** | 0.002 – 0.018 | **0.014** | 1.15 | Primary | 78 | **0.322** |  |
| Min <70%, Night 2 (min) | **0.0062** | 0.001 – 0.011 | **0.020** | 1.27 | Primary | 78 |  |  |
| Deep sleep %, Night 2 | −0.019 | −0.056 – 0.018 | 0.310 | 1.07 | Primary | 78 |  |  |
| Sleep score, Night 1 (0–3) | **0.552** | 0.122 – 0.982 | **0.013** | 1.08 | Primary | 78 |  |  |
| **Screen-based Model — Variables from univariate screen (Prism; n=78, R²=0.325, F(4,73)=8.80, p<0.0001; all VIF<1.3)** | | | | | | | |  |
| Min <70%, Night 1 (min) | **0.0098** | 0.002 – 0.018 | **0.018** | 1.17 | Screen | 78 | **0.325** |  |
| Min <70%, Night 2 (min) | **0.0062** | 0.001 – 0.011 | **0.018** | 1.25 | Screen | 78 |  |  |
| pRDI, Night 2 (events/hr) | 0.0083 | −0.006 – 0.022 | 0.248 | 1.07 | Screen | 78 |  |  |
| Sleep score, Night 1 (0–3) | **0.562** | 0.133 – 0.991 | **0.011** | 1.08 | Screen | 78 |  |  |
| *Notes: β = unstandardized regression coefficient. 95% CI by profile likelihood. Green=p<0.05. Residuals non-normally distributed across all specifications (expected for bounded integer AMS score). Primary model: a priori variable selection (Min<70% N1&N2, Deep Sleep% N2, Sleep Score N1). Screen model: variables with p<0.10 in univariate logistic screen.* | | | | | | | |  |
|  |  |  |  |  |  |  |  |  |
|  |  |  |  |  |  |  |  |  |
